# Supplementary material for: H-NGPCA: Hierarchical clustering of data streams with adaptive number of clusters and adaptive dimensionality
Source: PLoS One. 2026 Jan 5;21(1):e0339171. doi: 10.1371/journal.pone.0339171 (PMC12768385; doi:10.1371/journal.pone.0339171)
Supplement: Attachment — Submitted filename: review-PONE-D-25-43831.pdf [file pone.0339171.s001.pdf]

Review comments for PONE-D-25-43831 "H-NGPCA: Hierarchical clustering of data streams with adaptive number of clusters and adaptive dimensionality"

This paper proposes a new clustering algorithm, H-NGPCA, for data streams. It combines three ideas: hierarchical structure, centroid-based clustering algorithm (NG), and online principal component analysis within each cluster/unit. The proposed algorithm is parameter-free in clustering, meaning it can adaptively decide the number of clusters and the number of components in each cluster. Experimental results demonstrate its advantages over the state-of-the-art cluserting algorithm.

Overall, this paper is well presented. However, I have the following comments for improvement:

- The algorithm name "H-NGPCA" is used in most of the paper, while "HNGPCA" is used in in some places (e.g., the caption of Figure 2 and the discussion and conclusion section). Please, keep consistent.
- In equation (2), the meaning of m is unclear. While I get to know it is the number of eigenvalues from the latter, it is better to add an explanation after it directly.
- In the line following equation (3), $\check{\lambda}$ should be $\check{\lambda}_j$.
- It seems that μ may be missing from the second term on the right side of equations (15) and (16).
- Above equation (18), in the given small example, should "ten unit" and "one developed unit is replaced ..." be "ten outermost developed unit" and "one outermost developed unit is replaced ..."?
- In the bottom of page 9, check whether $Q^*_{C,j} < \phi Q_P$ is correct. And the equation is different from the one shown in Figure 7.
- On page 11 line 2: "...focus on significant extensions made by us in this work..." could simplify as "...focus on significant extensions in this work...".
- The text and the caption of Figure 11 refer to "two horizontal lines". Are they both referring to Fig 11.a? If so, only one horizontal line seems to be present in the figure. Also, Fig 11.b does not appeared to be discussed in the paper.
- The experiments compared clustering results with different algorithms. It claims that the adaptive dimensionality adjustment could "save a lot of computational effort". To support this claim, it would be helpful to include runtime results for comparison.
