# Supplementary material for: H-NGPCA: Hierarchical clustering of data streams with adaptive number of clusters and adaptive dimensionality
Source: PLoS One. 2026 Jan 5;21(1):e0339171. doi: 10.1371/journal.pone.0339171 (PMC12768385; doi:10.1371/journal.pone.0339171)
Supplement: Attachment — Submitted filename: Response to Reviewers.pdf [file pone.0339171.s002.pdf]

Response to Reviewer Comments

Manuscript ID: `PONE-D-25-43831`

Title: *H-NGPCA: Hierarchical clustering of data streams with adaptive number of clusters and adaptive dimensionality*

General Response

We thank the reviewers and editor for their careful reading of our manuscript, and for their constructive comments. We have revised the manuscript accordingly. Below, we provide a detailed response to each point raised.

Reviewer 1

This manuscript introduces H-NGPCA, a hierarchical clustering algorithm for data streams that adaptively determines both the number of clusters and their local dimensionality. The method combines centroid-based (Neural Gas), model-based (PCA), and hierarchical clustering in a fully online manner. The work is technically sound, well-motivated, and addresses an important challenge in streaming data analysis. The experimental evaluation is thorough, and the visualizations effectively illustrate the algorithm's behavior. However, several aspects require clarification and improvement to strengthen the contribution and ensure reproducibility.

Reviewer Comment:

The pseudo-code in Appendix B (Algorithm 2) is a valuable addition, but the main text does not sufficiently explain the flow of the algorithm. For example, the interaction between the tree traversal (line 6–16) and the update steps (line 17–27) is unclear. A high-level summary in Section 3.1 would help readers understand the end-to-end process before diving into details.

Response:

We agree with the reviewer comment and revised section 3.1 substantially to present a more thorough and precise explanation of the algorithm. We further enhanced the link between the provided pseudo code and the mathematical description in section 3.1.

Reviewer Comment:

The authors rightly note that many online algorithms lack implementations, so they compare against offline methods. However, this puts H-NGPCA at a disadvantage. To fairness, consider including a streaming variant of a classic method (e.g., Streaming K-Means) even if not adaptive. In addition, I suggest that the author discuss more about the application of the method. https://doi.org/10.1016/j.compbiomed.2023.107244 DOI: 10.1109/ACCESS.2020.2970838

Response:

Within our benchmark (Table 2), we already included non-adaptive streaming algorithms such as MiniBatchKMeans++. We agree that the current manuscript does not sufficiently discuss potential applications of our method. However, a detailed evaluation of applicability would require numerous additional experiments, which are beyond the scope of this work. We have added a paragraph outlining promising application scenarios as part of our future work discussion, with respect to the provided paper.

Reviewer Comment:

Table 1 and 2 show that H-NGPCA performs well, but the standard deviations are missing. Including these would help assess the stability of the results across multiple runs.

Response:

We agree that adding the standard deviations will provide a better understanding of the algorithmic stability across multiple runs. We therefore added a section to the appendix to address this comment. It has to be noted that some algorithms have standard deviations of zero on certain data sets. This usually happened when the respective algorithm was unable to cluster the data set in any of the runs, such as when an algorithm is generally badly performing on high-dimensional data sets.

Reviewer Comment:

The claim that H-NGPCA outperforms BIRCH and Affinity Propagation in some cases is supported, but the discussion should emphasize that these are offline methods. The online capability of H-NGPCA is a significant advantage that should be highlighted more clearly.

Response:

We agree that the online capability of H-NGPCA is a key advantage and have revised the discussion in section 5.2 accordingly.

Reviewer Comment:

The complexity analysis in Appendix E.3 is detailed and correct. The per-data-point complexity does not account for the dimensionality adjustment (Algorithm 1), which involves Gram-Schmidt and eigenvalue regression. This should be included in the analysis.

Response:

We agree that the Gram-Schmidt (i) and eigenvalue regression (ii) for Algorithm 1 are missing. (i) Importantly, the Gram-Schmidt is only performed when the dimensionality is increased. In the case when the PCA unit dimensionality m is increased from an existing set of eigenvectors m_{old}, we only orthogonalize the newly added Δm eigenvectors (Gram–Schmidt) against the already existing m_{old}. This leads to

$$O(n m_{\text{old}} \Delta m) \tag{1}$$

which is typically much smaller than a full recomputation $O(nm^2)$, especially when $\Delta m \ll m_{\text{old}}$.

(ii) The linear eigenvalue regression is only performed on the first m eigenvalues which scales linearly $O(m)$.

We adjusted the Appendix E.3 accordingly to cover the computational complexity of the two algorithmic components of the dimensionality adjustment algorithm.

Reviewer Comment:

The authors acknowledge that H-NGPCA cannot handle three collinear clusters (Fig. 12) and lacks a merge mechanism. These are significant limitations. A brief discussion of how a merge mechanism could be integrated (e.g., via a quality measure pruning branches) would strengthen the paper.

Response:

We agree and updated the future work section to suggest first ideas on how to overcome these two limitations.

Reviewer Comment:

The algorithm currently uses five hyperparameters. While this is fewer than many streaming algorithms, a table showing the values used for each dataset (or a justification for fixed values) would aid reproducibility.

Response:

For each data set the hyperparameters shown in Table 7. were used. We added an extra paragraph to increase the reproducibility.

Reviewer 2

The paper is well-structured and clearly written, presenting a timely and relevant study on H-NGPCA. The hierarchical extension is explained coherently and supported by appropriate experimental validation. Here are some suggestions to further enhance the paper.

Reviewer Comment:

Abstract: The abstract could be strengthened by more explicitly highlighting the novelty of the proposed method. Additionally, presenting key quantitative results would give readers a clearer and more concrete understanding of the contribution and its significance.

Response:

We revised the abstract to highlight the novelty of the proposed H-NGPCA algorithm including the presentation of quantitative results.

Reviewer Comment:

Introduction

- The introduction clearly outlines the problem statement; however, the review of related work is somewhat limited. A more thorough discussion of existing dimensionality reduction techniques (e.g., Kernel PCA, Autoencoders, Deep Learning–based methods, t-SNE, UMAP) is recommended. Furthermore, the manuscript should more explicitly articulate the advancement of H-NGPCA over conventional NGPCA, providing a stronger justification for the incorporation of the hierarchical structure.
- A comparison of these dimensionality reduction techniques, along with a clear justification of their relevance, would strengthen the discussion.
- To further strengthen the problem statement, it is advisable to include supporting references that validate and contextualize the identified research gap.

Response:

We agree that the state-of-the-art in its current form does not reflect deep learning based or non-linear dimensionality reduction methods in general. We added a discussion and supporting references to the related work section to close this gap. In addition, the advancements of H-NGPCA over the conventional NGPCA algorithm are highlighted in the introduction and the abstract.

Reviewer Comment:

Methodology

- The methodological description is detailed and logically presented, with adequate mathematical formalization.
- Parameter choices, computational aspects, and algorithmic flow are well explained, ensuring reproducibility.

Response:

We thank the reviewer for the positive feedback.

Reviewer Comment:

Results and Discussion

- The experimental evaluation is comprehensive, demonstrating clear improvements over conventional PCA and related methods.
- Results are supported by appropriate benchmarking and statistical validation.

Response:

We thank the reviewer for the positive feedback.

Reviewer Comment:

Conclusion

- The conclusion effectively summarizes the contributions and highlights the practical implications of the work.
- Future directions are appropriately suggested, adding value to the study.

Response:

We thank the reviewer for the positive feedback.

Reviewer 3

This paper proposes a new clustering algorithm, H-NGPCA, for data streams. It combines three ideas: hierarchical structure, centroid-based clustering algorithm (NG), and online principal component analysis within each cluster/unit. The proposed algorithm is parameter-free in clustering, meaning it can adaptively decide the number of clusters and the number of components in each cluster. Experimental results demonstrate its advantages over the state-of-the-art cluserting algorithm. Overall, this paper is well presented. However, I have the following comments for improvement:

Reviewer Comment:

The algorithm name "H-NGPCA" is used in most of the paper, while "HNGPCA" is used in in some places (e.g., the caption of Figure 2 and the discussion and conclusion section). Please, keep consistent.

Response:

We agree that it is important to use the algorithm name consistent throughout the work. We adjusted the corresponding errors accordingly.

Reviewer Comment:

In equation (2), the meaning of m is unclear. While I get to know it is the number of eigenvalues from the latter, it is better to add an explanation after it directly.

Response:

In equation (2) the unit specific learning rate $\epsilon_j(t)$ is calculated. Therefore, for each dimension m, the variance miss-match $\bar{\Gamma}_{j,i}(t)$ between the eigenvalues and data variance is used.

Lastly, the learning rate is obtained by averaging over all PCA dimensions m. We adjusted the corresponding paragraph around equation (2) to clarify the meaning of m.

Reviewer Comment:

In the line following equation (3), $\hat{\lambda}$ should be $\hat{\lambda}_j$.

Response:

We agree and corrected this mistake.

Reviewer Comment:

It seems that μ may be missing from the second term on the right side of equations (15) and (16)

Response:

We indeed forgot μ in both equation (15) and (16). The missing parameter was added to both equations.

Reviewer Comment:

Above equation (18), in the given small example, should ”ten unit” and ”one developed unit is replaced ...” be ”ten outermost developed unit” and ”one outermost developed unit is replaced ...”?

Response:

We agree, that in the provided small example the wording should include ”outermost”. We adjusted the the paragraph to reflect these changes.

Reviewer Comment:

In the bottom of page 9, check whether $Q^*_{C,j} < \psi Q_P$ is correct. And the equation is different from the one shown in Figure 7.

Response:

The equation shown in Figure 7 is indeed wrong and we adjusted the figure to match the correct formula described in the mentioned paragraph.

Reviewer Comment:

On page 11 line 2: ”...focus on significant extensions made by us in this work...” could simplify as ”...focus on significant extensions in this work...”.

Response:

We agree and adjusted the corresponding paragraph.

Reviewer Comment:

The text and the caption of Figure 11 refer to "two horizontal lines". Are they both referring to Fig 11.a? If so, only one horizontal line seems to be present in the figure. Also, Fig 11.b does not appeared to be discussed in the paper.

Response:

Both horizontal lines are referring to Fig 11.a. We noticed, that due to the y-axis scaling on this $m = 1024$ dimensional data set, both lines are visually overlapping. We therefore removed them from the image, and instead provided the numbers directly in the corresponding paragraph. Further, we added the discussion on Fig 11.b, which appears to be missing.

Reviewer Comment:

The experiments compared clustering results with different algorithms. It claims that the adaptive dimensionality adjustment could "save a lot of computational effort". To support this claim, it would be helpful to include runtime results for comparison.

Response:

The computational complexity for each data point presentation (Appendix E.3) shows the dependency of our algorithm on the PCA dimensionality m:

$$O(nmd + nm^2 + 2^{d-1}) \tag{2}$$

By controlling m for each unit individually and by starting with a minimal dimensionality $m(t = 0) = 2$, we greatly reduce the quadratic dependency on the unit dimensionality in the first and the linear dependency in the second term.
